# CircSmox knockdown alleviates PC12 cell apoptosis and inflammation in spinal cord injury by miR‐340‐5p/Smurf1 axis

**DOI:** 10.1002/iid3.824

**Published:** 2023-04-12

**Authors:** Ziyin Han, Zufang Mou, Yulong Jing, Rong Jiang, Tao Sun

**Affiliations:** ^1^ Department of Traumatic Orthopedics Yantaishan Hospital Yantai China; ^2^ Department of Nosocomial Infection Yantaishan Hospital Yantai China; ^3^ Department of Physiology Binzhou Medical University Binzhou China

**Keywords:** circSmox, lipopolysaccharide (LPS), miR‐340‐5p, PC12 cell, Smurf1, spinal cord injury

## Abstract

**Background:**

Spinal cord injury (SCI) is a traumatic central nervous system disorder that leads to irreversible neurological dysfunction. Emerging evidence has shown that differentially expressed circular RNAs (circRNAs) after SCI is closely associated with the pathophysiological process. Herein, the potential function of circRNA spermine oxidase (circSmox) in functional recovery after SCI was investigated.

**Methods:**

Differentiated PC12 cells stimulated with lipopolysaccharide (LPS) were employed as an in vitro model for neurotoxicity research. Levels of genes and proteins were detected by quantitative real‐time PCR and Western blot analysis. Cell viability and apoptosis were determined by CCK‐8 assay and flow cytometry. Western blot analysis was used to detect the protein level of apoptosis‐related markers. The levels of interleukin (IL)‐1β, IL‐6, IL‐8, and tumor necrosis factor (TNF)‐α. Dual‐luciferase reporter, RIP, and pull‐down assays were used to confirm the target relationship between miR‐340‐5p and circSmox or Smurf1 (SMAD Specific E3 Ubiquitin Protein Ligase 1).

**Results:**

LPS elevated the levels of circSmox and Smurf1, but decreased the levels of miR‐340‐5p in PC12 cells in a dose‐dependent manner. Functionally, circSmox silencing alleviated LPS‐induced apoptosis and inflammation in PC12 cells in vitro. Mechanistically, circSmox directly sponged miR‐340‐5p, which targeted Smurf1. Rescue experiments showed that miR‐340‐5p inhibition attenuated the neuroprotective effect of circSmox siRNA in PC12 cells. Moreover, miR‐340‐5p suppressed LPS‐triggered neurotoxicity in PC12 cells, which was reversed by Smurf1 overexpression.

**Conclusion:**

CircSmox enhances LPS‐induced apoptosis and inflammation via miR‐340‐5p/Smurf1 axis, providing an exciting view of the potential involvement of circSmox in SCI pathogenesis.

## Graphical abstract

CircSmox reinforces lipopolysaccharide‐induced neurotoxicity in PC12 cells in vitro.

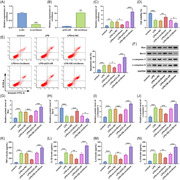

## 1 INTRODUCTION

Traumatic spinal cord injury (SCI) is one of the most devastating injuries, which can cause irreversible sensory deficit, neurological damage, dysfunction, and necrosis.^1^ SCI affects over 2.5 million people worldwide, and approximately 50%–80% of patients with SCI suffer from long‐term moderate to severe traumatic pain owing to the lack of effective management.^2^, ^3^ SCI is a two‐step process where the primary injury is followed by a progressive secondary injury characterized a cascade of biochemical and cellular processes involving the activation of neuroinflammation, proapoptotic signaling, vascular ischemia, cytotoxic debris, and lipid peroxidation.^4^, ^5^, ^6^ Thus, further investigation on the molecular mechanism underlying secondary injury are of great significance for developing effective strategy for SCI treatment.

**Table 1:** Primers sequences used for quantitative real‐time PCR (qRT‐PCR).

Name		Primers (5′−3′)
circSmox	Forward	TGCTACCTTACCAACCGTGG
	Reverse	CTGTCGCCACTGGATTCACA
Smox	Forward	GTGCGAGGATTGTGAGGTGA
	Reverse	CCCAAAAGGGCTCCTCGAAT
rno‐miR‐340‐5p	Forward	TCGGCAGGTTATAAAGCAATGA
	Reverse	CTCAACTGGTGTCGTGGAGT
Smurf1	Forward	GGACAACAGTGCAGGGACAA
	Reverse	TTCCCGACACTGTGCTTCTG
GAPDH	Forward	GCTCTCTGCTCCTCCCTGTTCTA
	Reverse	GCGCCCAATACGACCAAATC
U6	Forward	CTCGCTTCCGCAGCACAT
	Reverse	CTCGCTTCCGCAGCACAT

Circular RNAs (circRNAs) are one of noncoding RNAs with a covalently closed continuous loop that lacks 5′−3′ ends, thereby they are highly stable and can resistant to degradation by exonuclease RNase R.^7^ Research increasingly reported that circRNAs play a significant potential role in modulating diverse crucial biological processes related to carcinogenesis, metabolism, and inflammation.^8^, ^9^, ^10^ Moreover, the dysregulated expression of circRNAs has been observed in various diseases, such as cancer, immune system diseases, cardiovascular disease, and nervous system disease, and broadly participate in the pathogenesis and development of these diseases.^11^, ^12^, ^13^, ^14^ Besides that, studies have also showed that differential expression of circRNAs after SCI is tightly associated with the pathophysiological process.^15^ CircRNA spermine oxidase (circSmox, ID: rno‐Smox_0001) is originated from Smox gene in chr3: 124068796 | 124102267, which was identified to be highly expressed in the rat spinal cord following SCI.^16^ However, the function of circSmox in pathogenesis of SCI remains vague.

Herein, the potential function and molecular mechanism of circSmox in SCI pathological process was studied in vitro by treating differentiated PC12 cells, which are widely used as the model of neurons in vitro,^17^ with the lipopolysaccharide (LPS), which may provide a novel insight into the pathogenesis of SCI.

## 2 MATERIALS AND METHODS

### 2.1 Cell culture and treatment

Undifferentiated PC12 cells were purchased from American Type Culture Collection (Cat#CRL‐1721, ATCC) and grown in RPMI‐1640 medium (Cat#30‐2001, ATCC) containing 5% fetal bovine serum (FBS) (Cat# 30‐2020, ATCC), 1% antibiotics (streptomycin/penicillin) (Cat#30‐2300, ATCC) and 10% heat‐inactivated horse serum (Cat#26050070, Thermo Fisher Scientific) with 5% CO_2_ atmosphere at 37°C. After attaching to culture plates, the medium was changed to differentiation medium (RPMI‐1640 supplemented with 50 ng/mL nerve growth factor [NGF] [Cat#N0513, Sigma‐Aldrich]), 1% horse serum (Cat#12449c, Sigma‐Aldrich), and 1% antibiotics (Cat#30‐2300, ATCC) for 7 days to obtain neuronal differentiated PC12 cells. All subsequent experiments were conducted with differentiated PC12 cells.

PC12 cells were stimulated with increasing doses of LPS (0, 1, 2, 5, or 10 μg/mL, Cat#L2630, Sigma‐Aldrich) for 12 h. 5 μg/mL was selected to mimic the model of neuron injury in PC12 cells in vitro, cells treated with same volume of phosphate buffer saline (PBS, Cat#P4474, Sigma‐Aldrich) were used as the control, and cells were differentiated before experimental treatments.

### 2.2 Quantitative real‐time PCR (qRT‐PCR)

The TRIzol reagent (Cat#15596026, Invitrogen) was employed for the preparation of total RNA. Approximately 3 µg of total RNAs isolated from cultured PC12 cells were incubated with 3 U/μg of RNase R (Cat# R4875, Sigma‐Aldrich) or Mock without enzymatic activity for 1 h at 37°C. The PrimeScript RT Reagent Kit (Cat#RR037B, Takara) was used to reverse transcribe the RNA sample into cDNA, and then qRT‐PCR analysis was conducted with SYBR™ Green PCR Master Mix (Cat#4309155, Thermo Fisher Scientific) and primers (Table 1). PC12 cells were mixed with 5 μg/mL Actinomycin D (Cat# SBR00013, Sigma‐Aldrich) for 0, 6, 12, or 24 h to block transcription, and the half‐life of circSmox and linear Smox were measured using qRT‐PCR. The relative fold changes were represented by CT value with GAPDH (for circSmox, Smox, and Smurf1) or U6 (rno‐miR‐340‐5p) as an internal reference.^18^

### 2.3 Cell transfection

The pCD5‐ciR circSmox overexpressing plasmid (OE‐circSmox), pcDNA3.1 Smurf1 overexpressing plasmid (OE‐murf1), circSmox‐specific siRNA (si‐circSmox) and the negative control (NC) (pCD‐ciR, pcDNA, or si‐NC) were synthesized by GeneCopoepia Biosciences. The mimic or inhibitor of miR‐340‐5p and mimic or inhibitor control (miR‐NC or anti‐miR‐NC) were provided by Ribobio. Then PC12 cells were seeded into a 24‐well plate and transiently transfected with 50 nM of miRNA mimic, inhibitor or negative controls, or 100 nM of si‐circSmox or si‐NC, or 100 ug of OE‐circSmox, OE‐ murf1 or negative controls using Lipofectamine 2000^19^ (Cat#11668019, Invitrogen). After 48 h of transfection, cell were subjected to LPS treatment for subsequent analysis.

### 2.4 Cell counting kit 8 (CCK‐8) assay

PC12 cells were cultured on 96‐well plates overnight and subjected assigned transfection. Forty‐eight later, cells were exposed to 5 μg/mL LPS for 12 h. Subsequently, 10 μL of CCK‐8 solution (Cat#C003, Beyotime) was added and incubated for 3 h. Finally, the optical density was measured at 450 nm to calculate cell viability.^20^

### 2.5 Flow cytometer

Transfected PC12 cells subjected to 5 μg/mL LPS treatment for 12 h were washed with PBS and trypsin to obtain the single‐cell suspensions. Then cell were fixed in ice‐cold 70% ethanol, followed by the mixture with 5 μL FITC‐AnnexinV and 5 μL propidium iodide (PI) (Cat# 56570, BD Biosciences) for 20 min under darkness. Apoptosis was examined by a FACScan flow cytometry (BD Biosciences).^21^

### 2.6 Western blot analysis

PC12 cells were lysed in precooled RIPA lysis buffer (Cat# P0013B, Beyotime) containing 1% phenylmethanesulfonyl fluoride (PMSF), and protein concentration was qualified by a BCA method (Cat#P0009, Beyotime). Then equal amounts of protein were separated by 10% SDS‐PAGE, and transferred to PVDF membranes (Cat#PVH00010, Millipore, Darmstadt, Germany). After sealing with 5% nonfat powdered milk for 2 h, primary incubation was performed overnight at 4°C with primary antibodies at a dilution of 1:1000. And then membranes were probed with an anti‐rabbit or anti‐mouse HRP‐conjugated second antibody at 37°C for 2 h following washing three times with TBST. Protein bands were observed by ECL detection reagent (Cat# PE0010, Solarbio).^22^ The densitometry of the gel bands was analyzed using ImageJ (National Institutes of Health). The primary antibodies included: Bcl‐2 (ab194583), Bax (ab32503), Cleaved caspase 3 (c‐caspase 3) (ab2302), Smurf1 (ab57573), Cleaved Caspase‐9 (c‐caspase 9) (ab2324), and GAPDH (ab181602), all obtained from Abcam.

### 2.7 Enzyme‐linked immunosorbent assay (ELISA)

The cell culture supernatant of transfected PC12 cells underwent 5 μg/mL LPS treatment for 12 h were collected by centrifugation at 12,000x*g* for 10 min at 4°C and the levels of tumor necrosis factor‐α (TNF‐α) (ab100785), interleukin‐6 (IL‐6) (ab234570), IL‐1β (ab100768) (Abcam) and IL‐8 (Cat#RAB1147, Sigma‐Aldrich) were detected by using the corresponding commercial ELISA kits according to the manufacturer's instructions.

### 2.8 Dual‐luciferase reporter assay

The fragments of circSmox and Smurf1 3′UTR covering the miR‐340‐5p binding sites and the point mutated sequences in target sites were amplified and inserted into the pmirGLO report luciferase vector (Cat#E1330, Promega) to establish wild‐type (WT) or mutated reporter vectors (WT‐circSmox/Smurf1 3′UTR or MUT‐circSmox/Smurf1 3′UTR). Then PC12 cells were seeded in a 24‐well plate and cotransfected with constructed luciferase vector, and miR‐340‐5p or miR‐NC. 48 h later, luciferase activity was detected using Dual‐Luciferase®Reporter Assay System (Cat# E1910, Promega).^23^

### 2.9 RNA immunoprecipitation (RIP) assay

The lysates of PC12 cells obtained by RIP lysis buffer were incubated with magnetic beads conjugated with AGO2 antibody or a negative IgG antibody at 4°C overnight (Cat#17‐704, Millipore). After the addition of proteinase K in the bead/antibody/lysate mixture, the enrichment of miR‐340‐5p and circSmox with AGO immunoprecipitation was detected by using qRT‐PCR.

### 2.10 RNA pull‐down assay

Biotinylated miR‐340‐5p probes (Bio‐miR‐340‐5p) or the control probes (Bio‐NC) were synthesized by Genepharma (Shanghai, China). PC12 cells infected with RNA probes were lysed and then incubated with streptavidin‐coupled magnetic beads (Cat# 65305, Thermo Fisher Scientific). After purification by TRIzol, the levels of circSmox was detected by qRT‐PCR.

### 2.11 Statistical analyses

Each experiment was independently repeated three times. The data in the bar graphs were manifested as mean ± standard deviation. Paired or unpaired *t*‐test, or Mann–Whitney test if not normally distributed was used for the comparison of datasets containing two groups. Statistical difference among multiple groups were conducted by analysis of variance (ANOVA) followed by Tukey's posttest. All statistical analysis was performed with GraphPad Prism 6 software (GraphPad), and *p* < .05 suggested significant differences (*p < .05, **p < .01. ***p < .001).

## 3 RESULTS

### 3.1 LPS treatment dose‐dependently increases circSmox expression in PC12 cells

**Figure 1:**
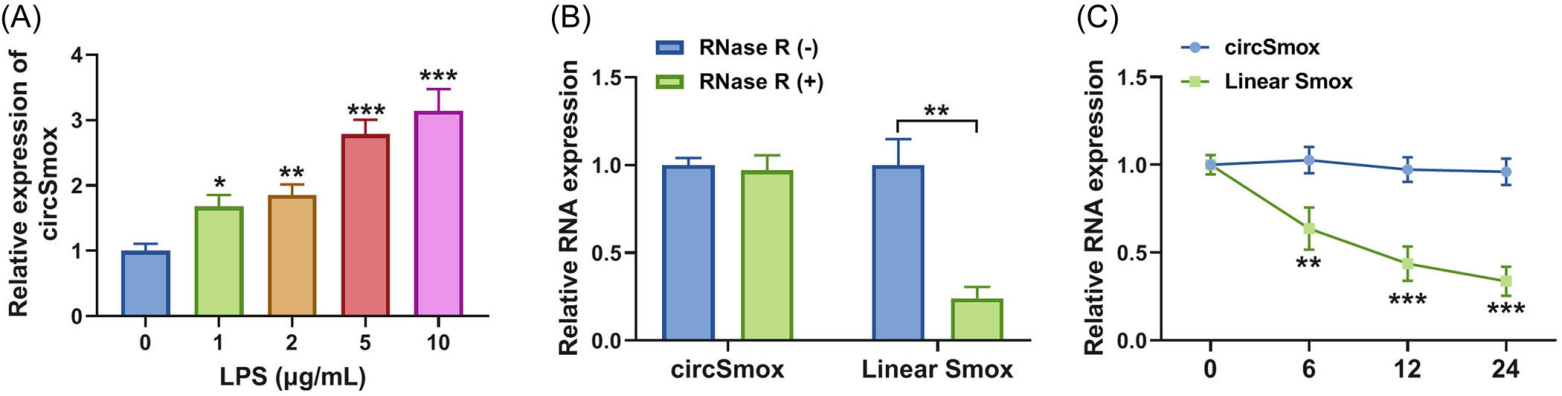
Lipopolysaccharide (LPS) treatment dose‐dependently increases circSmox expression in PC12 cells. (A) Quantitative real‐time PCR (qRT‐PCR) analysis of circSmox expression in PC12 cells exposed to different concentrations of LPS (0, 1, 2, 5, or 10 μg/mL) for 12 h. (B) PC12 cells were treated with RNase R or Mock and the levels of circSmox and linear Smox were analyzed by qRT‐PCR. (C) The expression of circSmox and linear Smox was detected by qRT‐PCR in PC12 cells after Actinomycin D treatment. *p < .05, **p < .01, ***p < .001.

As shown in Figure 1A, exposure of PC12 cells to different concentrations of LPS (0, 1, 2, 5, or 10 μg/mL) for 12 h led to an increase of circSmox expression in a dose‐dependent manner, indicating the potential involvement of circSmox in LPS‐induced cytotoxicity. 5 μg/mL LPS was selected for subsequent experiment due to the 50% increase. Thereafter, the circular characteristics of circSmox were analyzed. It was found that circSmox was resistant to the degradation by RNase R relative to the linear Smox in PC12 cells (Figure 1B). Moreover, the half‐life of circSmox exceeded 24 h, while that of linear Smox mRNA was about 6 h in PC12 cells (Figure 1C), further indicating circSmox is a stable circRNA.

### 3.2 CircSmox reinforces LPS‐induced neurotoxicity in PC12 cells in vitro

**Figure 2:**
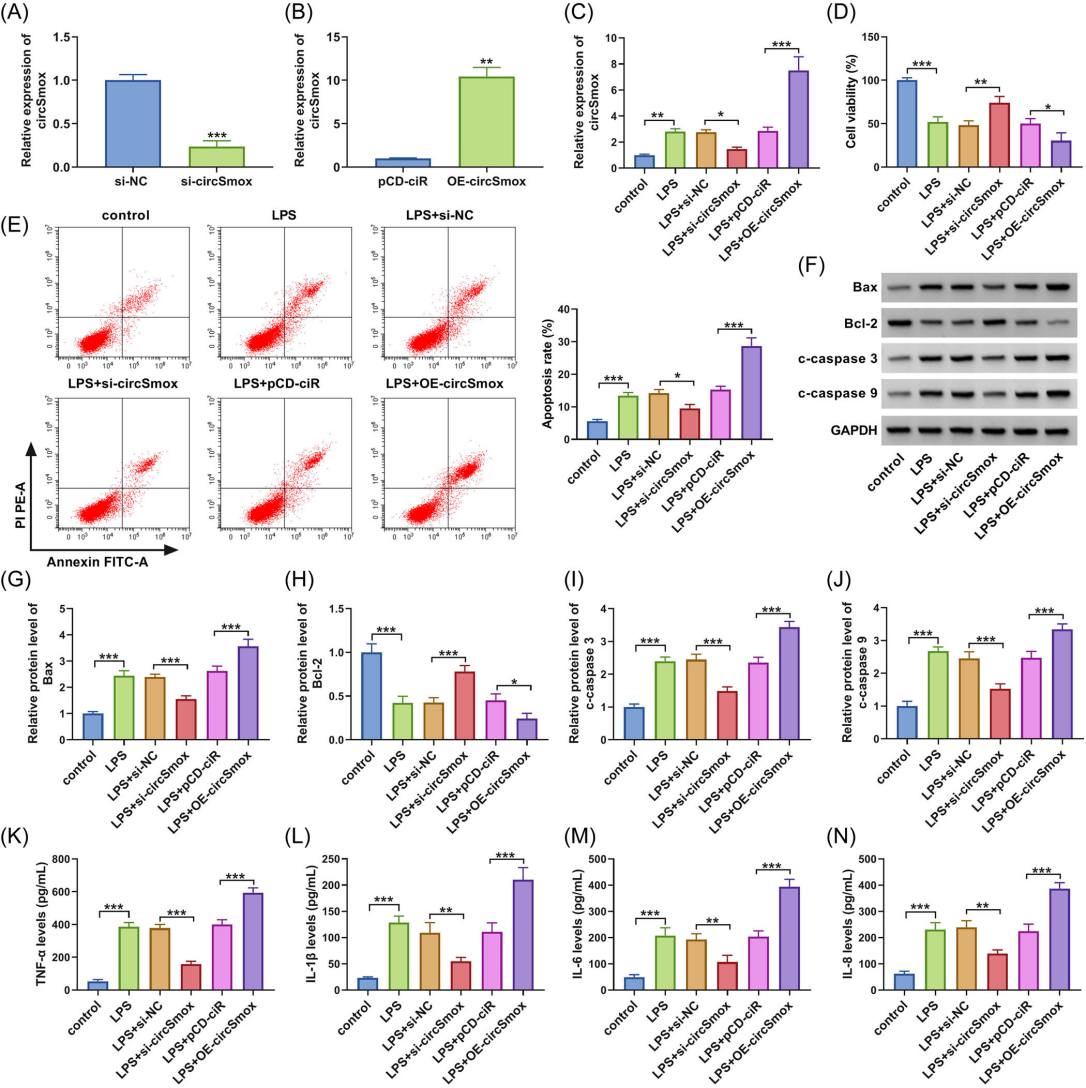
CircSmox reinforces lipopolysaccharide (LPS)‐induced neurotoxicity in PC12 cells in vitro. (A, B) The transfection efficiency of overexpression and the siRNA vectors of circSmox in PC12 cells using quantitative real‐time PCR (qRT‐PCR). (C–N) Transfected PC12 cells were treated with 5 μg/mL LPS for 12 h. (C) qRT‐PCR analysis of circSmox expression in PC12 cells. (D) CCK‐8 assay for cell viability. (E) Flow cytometry for cell apoptosis. (F–J) Western blot analysis analysis of the levels of Bax, Bcl‐2, c‐caspase 3, and c‐caspase 9 protein in cells. (K–N) ELISA analysis for the levels of tumor necrosis factor (TNF)‐α, interleukin (IL)‐1β, IL‐6, and IL‐8 in cells. *p < .05, **p < .01, ***p < .001.

Subsequently, to investigate the detailed functions of circSmox in LPS‐induced neurotoxicity, we constructed the overexpression and the siRNA vectors of circSmox (OE‐circSmox and si‐circSmox). The results of qRT‐PCR exhibited that circSmox was significantly downregulated or upregulated in PC12 cells transfected with si‐circSmox or OE‐circSmox compared with the cells transfected with si‐NC or pCD‐ciR (Figure 2A,B). In addition, as shown in Supporting Information: Figure S1, the viability of PC12 cells stimulated with 5 μg/mL LPS decreased by nearly 50%, thus, 5 μg/mL was selected to mimic the model of neuron injury in PC12 cells in vitro. Then transfected PC12 cells were treated with 5 μg/mL LPS for 12 h, and the elevation of circSmox caused by LPS was decreased by circSmox knockdown, but increased by circSmox overexpression in PC12 cells (Figure 2C). Functionally, the results in Figure 2D show that LPS treatment could decrease the viability of PC12 cells, which was rescued by circSmox knockdown, but reinforced by circSmox overexpression. Flow cytometry analysis suggested that circSmox knockdown reversed LPS‐induced apoptosis accompanying with the decreases of Bax, c‐caspase 3, and c‐caspase 9 as well as the increase of Bcl‐2, while circSmox overexpression showed opposite effects to enhance LPS‐induced apoptosis by elevating the levels of Bax, c‐caspase 3, and c‐caspase 9 and reducing the level of Bcl‐2 in PC 12 cells (Figure 2E–J). Besides that, ELISA analysis suggested that LPS evoked inflammatory response in PC12 cells, evidenced by the increases of TNF‐α, IL‐1β, IL‐6, and IL‐8, and when transfected with circSmox overexpression plasmids, this inflammatory response of PC12 cells was significantly increased, while transfection with circSmox siRNA decreased this inflammatory response in PC12 cells (Figure 2K–N). Taken together, these results suggested that circSmox knockdown had a neuroprotective effect.

### 3.3 CircSmox acts as a sponge for miR‐340‐5p

**Figure 3:**
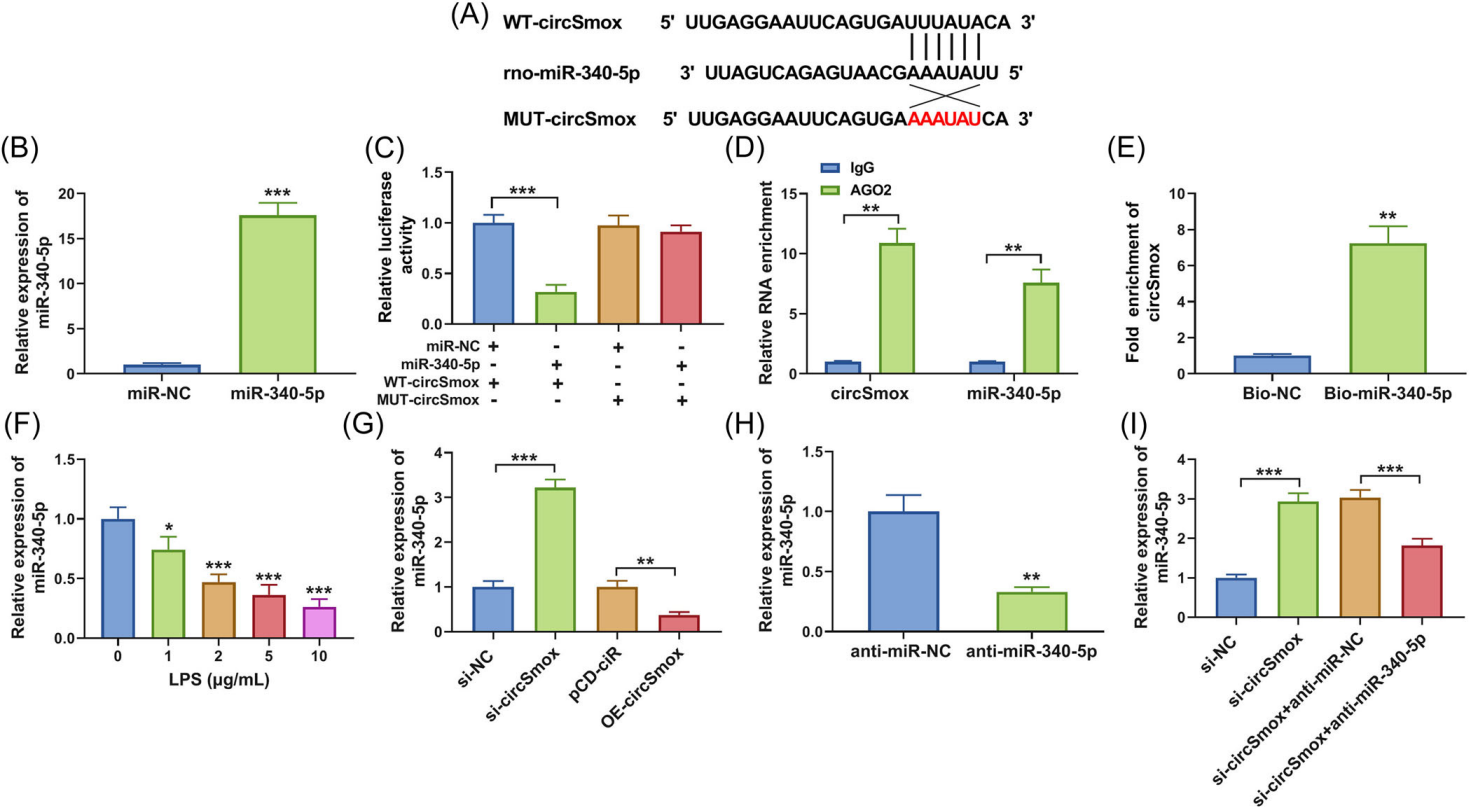
CircSmox acts as a sponge for miR‐340‐5p. (A) The putative conserved target site of miR‐340‐5p on circSmox was predicted by circAtlas database. (B) The transfection efficiency of miR‐340‐5p or miR‐NC in PC12 cells was validated by quantitative real‐time PCR (qRT‐PCR). (C) Dual‐luciferase reporter assay for the luciferase activity of wild‐type and mutated circSmox reporter after miR‐340‐5p overexpression in PC12 cells. (D) RNA immunoprecipitation (RIP) assay was executed in PC12 cells, and levels of circSmox and miR‐340‐5p were examined by qRT‐PCR. (E) RNA pull‐down with a biotinylated miR‐340‐5p probe was conducted in PC12 cells and the enrichment of circSmox was analyzed by qRT‐PCR. (F) qRT‐PCR analysis of miR‐340‐5p expression in PC12 cells exposed to different concentrations of LPS (0, 1, 2, 5, or 10 μg/mL) for 12 h. (G) qRT‐PCR analysis of miR‐340‐5p expression in PC12 cells transfected with OE‐circSmox, si‐circSmox, or the corresponding negative control. (H) The knockdown efficiency of anti‐miR‐340‐5p or anti‐miR‐NC was verified in PC12 cells using qRT‐PCR. (I) qRT‐PCR analysis of miR‐340‐5p expression in PC12 cells transfected with si‐NC, si‐circSmox, si‐circSmox + anti‐miR‐NC or si‐circSmox + anti‐miR‐340‐5p. *p < .05, **p < .01, ***p < .001.

Based on the competing endogenous RNA (ceRNA) hypothesis,^24^ the underlying miRNA of circSmox in PC12 cells were probed using bioinformatics analysis based on circAtlas database, and miR‐340‐5p was predicted to have the putative conserved target site on circSmox (Figure 3A). After confirming the transfection efficiency of miR‐340‐5p mimic (Figure 3B), the dual‐luciferase reporter assay was performed. The results showed that miR‐340‐5p mimic significantly reduced the luciferase activity of the wild‐type circSmox reporter vector, but not the mutated one in PC12 cells (Figure 3C). RIP assay was then conducted, it was found that the levels of circSmox and miR‐340‐5p were effectively higher in AGO immunoprecipitation than those in the IgG negative control in PC12 cells (Figure 3D). Moreover, RNA pull‐down assay in PC12 cells showed that circSmox was significantly captured by the biotinylated miR‐340‐5p probe with markedly enhanced fold‐changes (Figure 3E). All these data confirmed the binding between circSmox and miR‐340‐5p. Thereafter, we found that miR‐340‐5p expression was dose‐dependently decreased by LPS treatment in PC12 cells (Figure 3F). The level of miR‐340‐5p was downregulated by circSmox overexpression but upregulated by circSmox knockdown in PC12 cells (Figure 3G). Then the knockdown efficiency of miR‐340‐5p inhibitor was validated by qRT‐PCR analysis (Figure 3H), and we proved that miR‐340‐5p inhibitor overtly reduced circSmox knockdown‐triggered elevation of miR‐340‐5p in PC12 cells (Figure 3I). In all, these results confirmed that circSmox directly sponged miR‐340‐5p in PC12 cells.

### 3.4 MiR‐340‐5p knockdown reverses the neuroprotective effects of circSmox siRNA in PC12 cells

**Figure 4:**
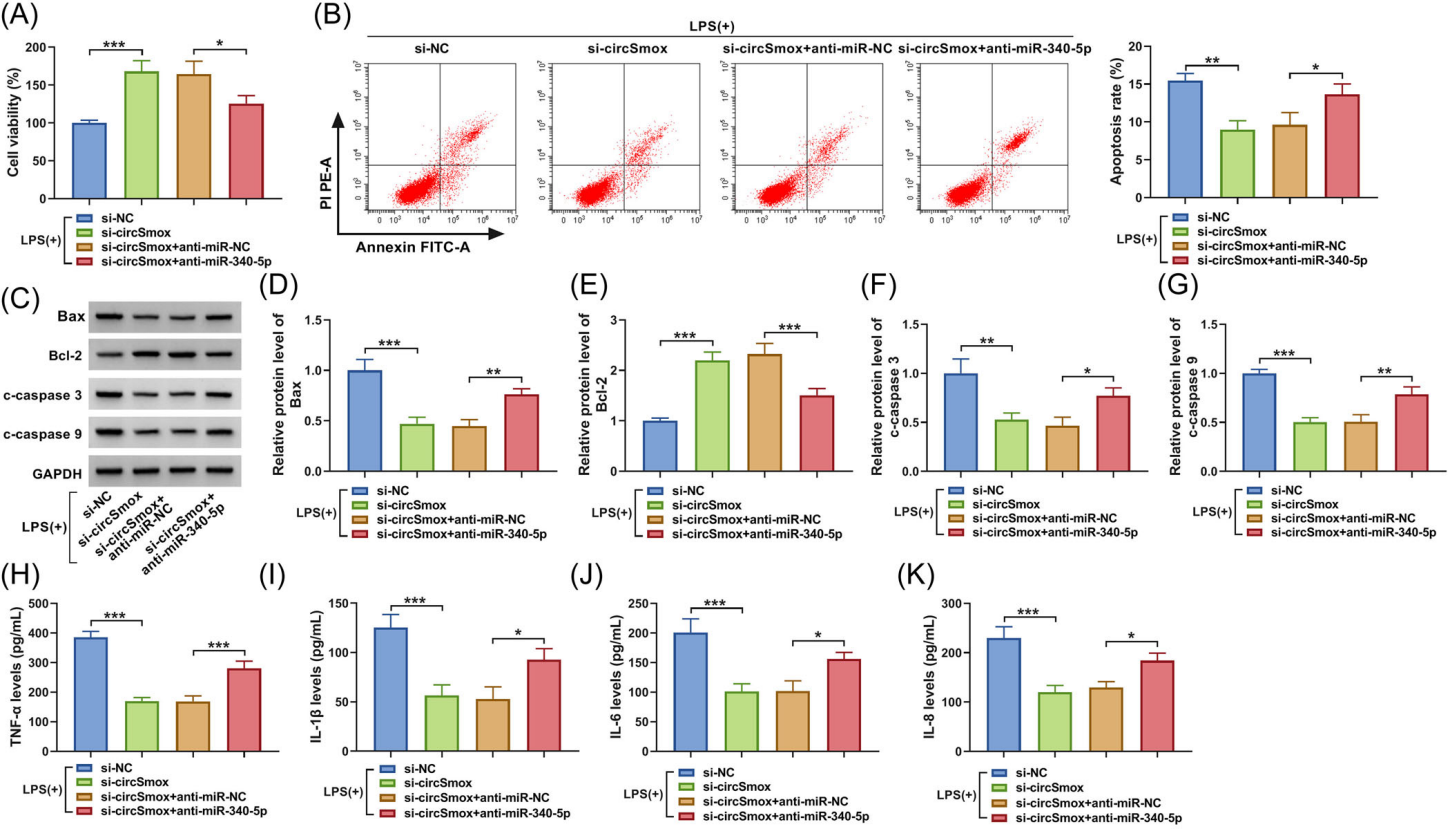
MiR‐340‐5p knockdown reverses the neuroprotective effects of circSmox siRNA in PC12 cells. (A–K) PC12 cells were transfected with si‐NC, si‐circSmox, si‐circSmox + anti‐miR‐NC, or si‐circSmox + anti‐miR‐340‐5p, and then treated with 5 μg/mL lipopolysaccharide (LPS) for 12 h. (A) CCK‐8 assay for cell viability. (B) Flow cytometry for cell apoptosis. (C–G) Western blot analysis analysis of the levels of Bax, Bcl‐2, c‐caspase 3, and c‐caspase 9 protein in cells. (H–K) ELISA analysis for the levels of tumor necrosis factor (TNF)‐α, interleukin (IL)‐1β, IL‐6, and IL‐8 in cells. *p < .05, **p < .01, ***p < .001.

To probe whether circSmox plays its biological role via circSmox/miR‐340‐5p axis, we performed a series of rescue experiments. The results revealed that inhibition of miR‐340‐5p counteracted circSmox knockdown‐evoked viability enhancing role (Figure 4A) and the inhibitory impacts on apoptosis (Figure 4B–G) and inflammatory response (Figure 4H–K) in PC12 cells in the presence of LPS. Collectively, these results demonstrated that circSmox might function as a sponge for miR‐340‐5p to contribute to LPS‐induced neurotoxicity in PC12 cells.

### 3.5 Smurf1 is a target of miR‐340‐5p in PC12 cells, and circSmox can regulate Smurf1 by sponging miR‐340‐5p

**Figure 5:**
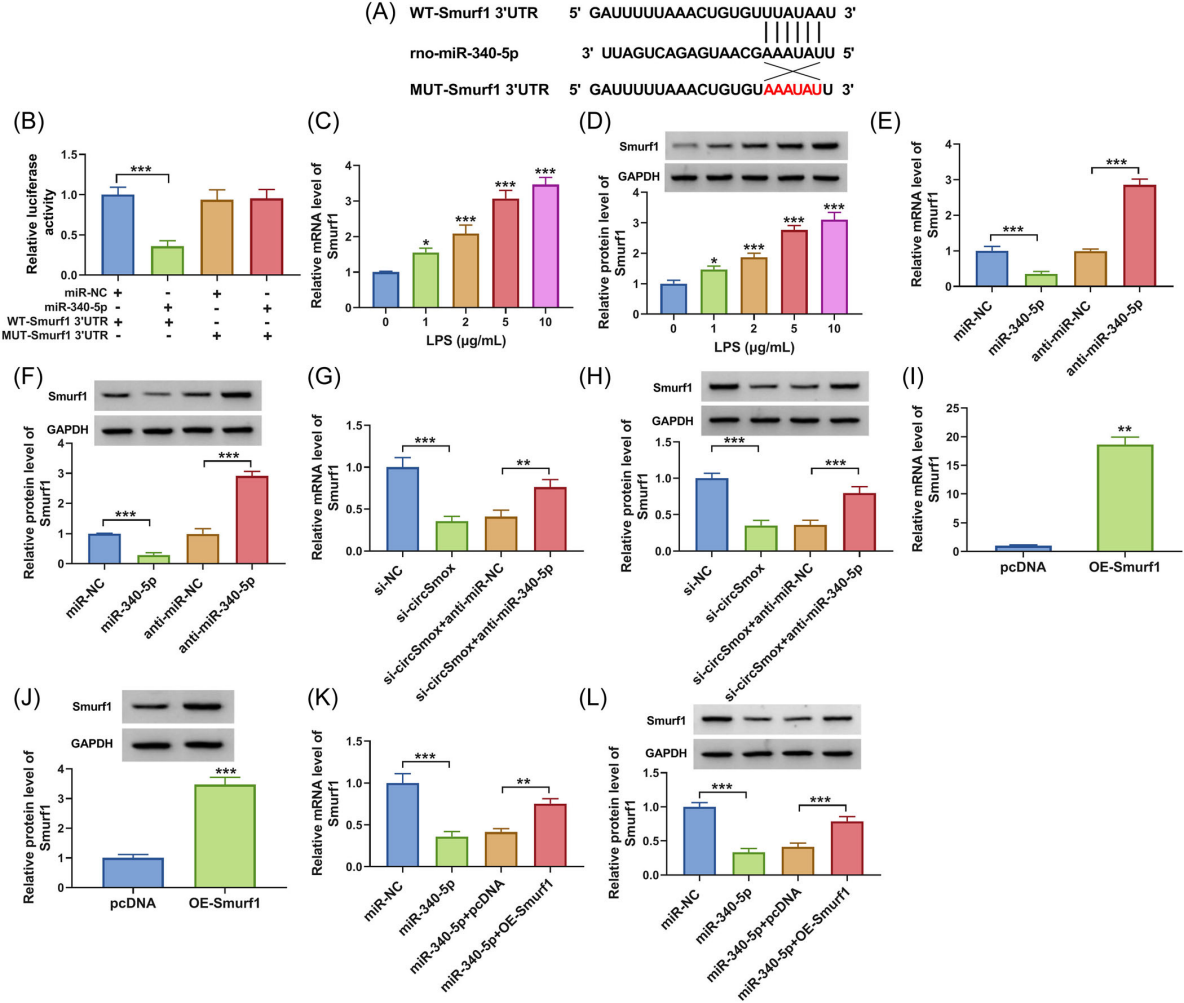
Smurf1 is a target of miR‐340‐5p in PC12 cells, and circSmox can regulate Smurf1 by sponging miR‐340‐5p. (A) The putative conserved target site of miR‐340‐5p on Smurf1 was predicted by TargetScan database. (B) Dual‐luciferase reporter assay for the luciferase activity of wild‐type and mutated Smurf1 reporter after miR‐340‐5p overexpression in PC12 cells. (C, D) Quantitative real‐time PCR (qRT‐PCR) and western blot analysis analysis for the levels of Smurf1 in PC12 cells exposed to different concentrations of LPS (0, 1, 2, 5, or 10 μg/mL) for 12 h. (E, F) qRT‐PCR and western blot analysis analysis for the levels of Smurf1 in PC12 cells transfected with miR‐340‐5p mimic, inhibitor or corresponding negative controls. (G, H) qRT‐PCR and western blot analysis analysis for the levels of Smurf1 in PC12 cells transfected with si‐NC, si‐circSmox, si‐circSmox + anti‐miR‐NC or si‐circSmox + anti‐miR‐340‐5p. (I, J) The transfection efficiency of OE‐Smurf1 or pcDNA was detected using qRT‐PCR and western blot analysis analysis. (K, L) qRT‐PCR and western blot analysis analysis of Smurf1 levels in PC12 cells transfected with miR‐NC, miR‐340‐5p, miR‐340‐5p + pcDNA, or miR‐340‐5p +;OE‐Smurf1. *p < .05, **p < .01, ***p < .001.

To explore the molecular mechanism underlying miR‐340‐5p, we then predicted the potential targets of miR‐340‐5p using TargetScan database. The results indicated that miR‐340‐5p had a putative conserved target site on Smurf1 (Figure 5A). Thereafter, results of dual‐luciferase reporter assay showed that miR‐340‐5p overexpression markedly reduced the luciferase activity of the wild‐type Smurf1 reporter, but failed to affect the mutated one in PC12 cells (Figure 5B). Smurf1 was found to be increased by LPS treatment in a dose‐dependent manner both at mRNA and protein levels (Figure 5C,D). Besides that, Smurf1 expression was decreased by miR‐340‐5p overexpression, but increased by miR‐340‐5p inhibition in PC12 cells (Figure 5E,F). All these results suggested that miR‐340‐5p targeted Smurf1 and negatively regulated its expression. Moreover, western blot analysis showed that circSmox knockdown or overexpression led to a decrease or an increase of Smurf1 in PC12 cells under LPS treatment (Supporting Information: Figure S2), besides that, we observed that knockdown of circSmox resulted in a decrease of Smurf1 expression in PC12 cells, which was rescued by the inhibition of miR‐340‐5p (Figure 5G,H). In addition, when we validated the transfection efficiency of Smurf1 overexpression plasmids (Figure 5I,J), it was proved that Smurf1 transfection rescued miR‐340‐5p mimic‐induced decrease of Smurf1 in PC12 cells (Figure 5K,L). Thus, we identified a circSmox/miR‐340‐5p/Smurf1 axis in PC12 cells.

### 3.6 MiR‐340‐5p attenuates LPS‐induced neurotoxicity in PC12 cells via Smurf1

**Figure 6:**
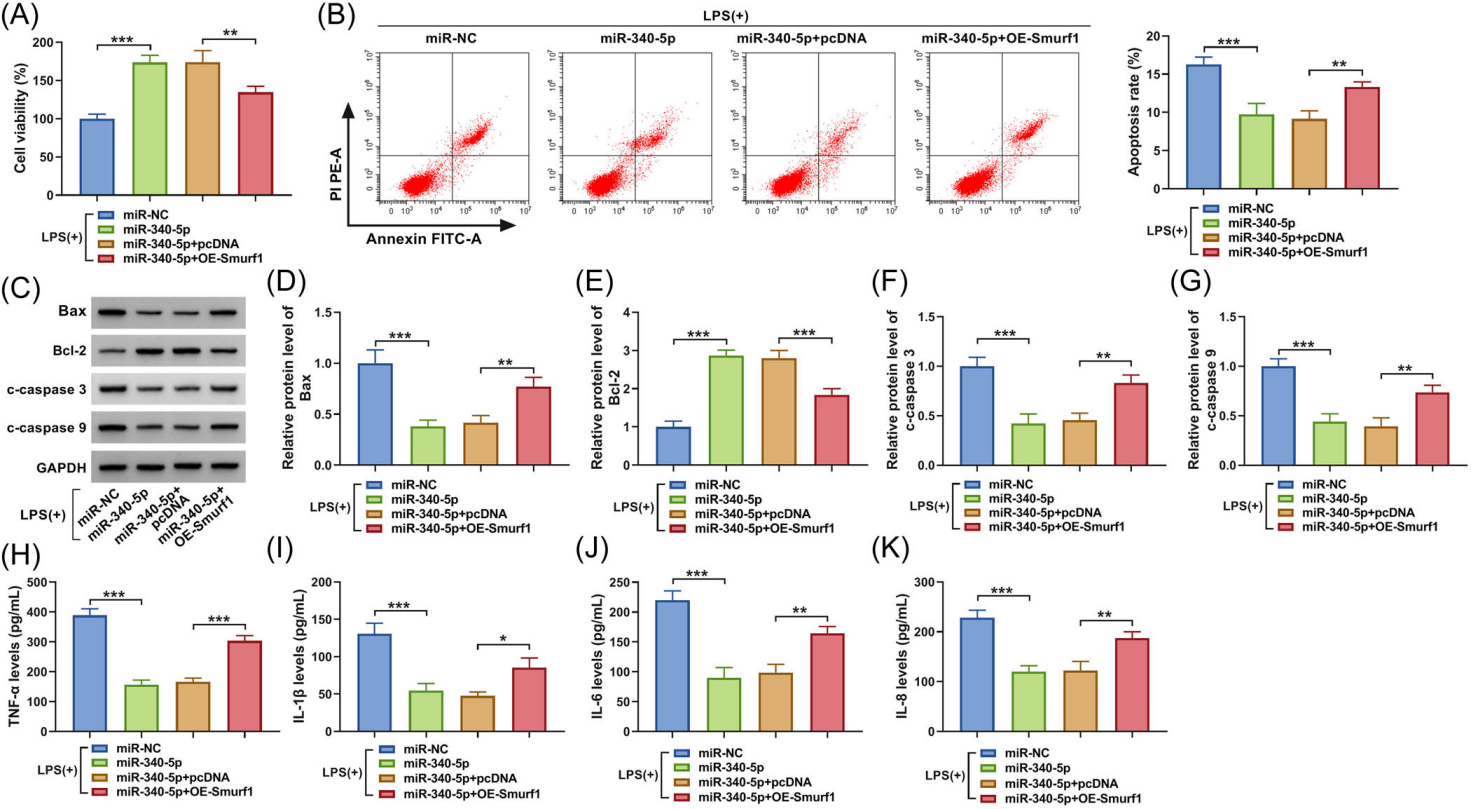
MiR‐340‐5p attenuates lipopolysaccharide (LPS)‐induced neurotoxicity in PC12 cells via Smurf1. (A–K) PC12 cells were transfected with miR‐NC, miR‐340‐5p, miR‐340‐5p + pcDNA, or miR‐340‐5p + OE‐Smurf1, and then treated with 5 μg/mL LPS for 12 h. (A) CCK‐8 assay for cell viability. (B) Flow cytometry for cell apoptosis. (C–G) Western blot analysis analysis of the levels of Bax, Bcl‐2, c‐caspase 3, and c‐caspase 9 protein in cells. (H–K) ELISA analysis for the levels of tumor necrosis factor (TNF)‐α, interleukin (IL)‐1β, IL‐6, and IL‐8 in cells. *p < .05, **p < .01, ***p < .001.

Next, the functions of miR‐340‐5p/Smurf1 axis in LPS‐induced neurotoxicity in PC12 cells were investigated. We found that miR‐340‐5p overexpression reversed LPS‐induced viability arrest, while this effect mediated by miR‐340‐5p was abolished by Smurf1 upregulation (Figure 6A). Besides, miR‐340‐5p mimic suppressed apoptosis in PC12 cells under LPS treatment, reflected by the decreased apoptosis rate, and protein levels of Bax, c‐caspase 3, and c‐caspase 9 as well as the increase level of Bcl‐2, which were abated by Smurf1 upregulation (Figure 6B–G). Moreover, the decreases of TNF‐α, IL‐1β, IL‐6, and IL‐8 levels in LPS‐treated PC12 cells caused by miR‐340‐5p were rescued by Smurf1 upregulation (Figure 6H–K). Altogether, miR‐340‐5p had a neuroprotective effect by repressing Smurf1.

## 4 DISCUSSION

Currently, the main clinical treatments for SCI are surgical procedures and high‐dose methylprednisolone, which are largely limited to prolong the survival rate of patients, but not recover the injured nerve functions.^25^ Thus, in‐depth understanding the basic neurobiology of SCI is necessary for the development of available strategies for SCI therapy. At present, a large number of findings suggest that circRNAs play key roles in human diseases and exhibit great potential as biomarkers and therapeutic targets.^26^ In SCI, some circRNAs have also been recognized to be involved in the pathogenesis by regulating the secondary damage. For example, circRNA‐2960 was demonstrated to be increased in SCI rats, and induced apoptosis and inflammation at the lesion site by targeting miR‐124.^27^ Circ_0000962 was found to have a neuroprotective effect by activating PI3K/Akt and blocking of NF‐κB via miR‐302b‐3p to suppress inflammatory response in vitro cell model of SCI.^28^ Additionally, Sun et al. showed that circTYW1 contributed to functional recovery in rats after SCI and suppressed apoptosis in the lesion site as well as oxygen‐glucose deprivation (OGD)‐induced PC12 cells by activating ERK1/2 signaling through miR‐380/FGF9 axis.^29^ Therefore, circRNAs are also have roles in function recovery after SCI. In this study, an increased expression of circSmox was discovered in LPS‐induced PC12 cells. Functionally, disrupting circSmox reversed LPS‐evoked inflammatory response and apoptosis in PC12 cells, while circSmox overexpression showed opposite effects, indicating the neurotoxic role of circSmox in PC12 cells.

CircRNAs can serve as a ceRNA to prevent miRNA‐mediated degradation of its downstream gene through sponging shared miRNAs.^24^, ^30^ Therefore, the underlying miRNA/mRNA network of circSmox was then investigated. This study confirmed that circSmox directly bound to miR‐340‐5p, which targeted Smurf1. Moreover, circSmox could regulate Smurf1 expression by miR‐340‐5p, suggesting the circSmox/miR‐340‐5p/Smurf1 axis. Gao et al. showed that miR‐340‐5p overexpression could reduce neuropathic pain and inflammation in by rat models after chronic constriction injury by regulating Rap1A. Moreover, miR‐340‐5p re‐expression induced locomotor function recovery in rat models after SCI and relieved neuroinflammation, oxidative stress, and apoptosis via blocking P38‐MAPK signaling. Smurf1 has been shown to be increased in spinal cord of rats after SCI.^31^ Disrupting Smurf1 expression ameliorated LPS‐evoked neuroinflammation and neuronal necroptosis in PC12 cells.^32^ Besides that, Zhao's team showed that Smurf1 attenuated miR‐125b‐meidated neurological recovery after SCI by promoting KLF2 degradation.^33^ All these data indicated the neurotoxic action of Smurf1 in SCI. In the present study, we found that LPS decreased miR‐340‐5p expression, but elevated Smurf1 expression in PC12 cells. MiR‐340‐5p also showed a neuroprotective effect by suppressing LPS‐induced apoptosis and inflammation in PC12 cells, which were reversed by Smurf1 upregulation. Importantly, miR‐340‐5p inhibition abolished the neuroprotective action of circSmox siRNA in LPS‐stiluated PC12 cells.

**Figure 7:**
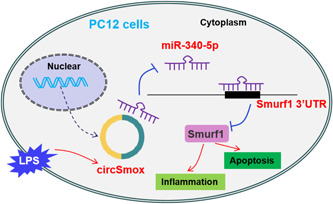
CircSmox mediates lipopolysaccharide (LPS)‐induced inflammation and apoptosis in PC12 cells via miR‐340‐5p/Smurf1 axis.

In conclusion, this work for the first time confirmed that circSmox contributed to LPS‐induced neuroinflammation and apoptosis in PC12 cells via miR‐340‐5p/Smurf1 axis (Figure 7), suggesting a novel insight into the mechanisms of secondary injury in SCI and a new target for SCI therapy.

## AUTHOR CONTRIBUTIONS

Zufang Mou and Yulong Jing designed and performed the research; Rong Jiang and Tao Sun analyzed the data; Ziyin Han wrote the manuscript. All authors read and approved the final manuscript.

## CONFLICT OF INTEREST STATEMENT

The authors declare no conflict of interest.

## Supporting information


Figure S1 The viability of PC12 cells after lipopolysaccharide (LPS) treatment. CCK‐8 assay for cell viability after exposing to different concentrations of LPS (0, 1, 2, 5, or 10 μg/mL) for 12 h. *p < .05, **p < .01, ***p < .001.

Figure S2 The expression of Smurf1 in lipopolysaccharide‐treated PC12 cells after circRNA spermine oxidase overexpression or knockdown. Western blotting analysis of Smurf1 in PC12 cells after circSmox overexpression or knockdown. ***p < .001.

## Data Availability

Data sharing is not applicable to this article as no datasets were generated or analyzed during the current study.
