# Supplementary figures and images for: CircSmox knockdown alleviates PC12 cell apoptosis and inflammation in spinal cord injury by miR‐340‐5p/Smurf1 axis
Source: Immun Inflamm Dis. 2023 Apr 12;11(4):e824. doi: 10.1002/iid3.824 (PMC10091371; doi:10.1002/iid3.824)

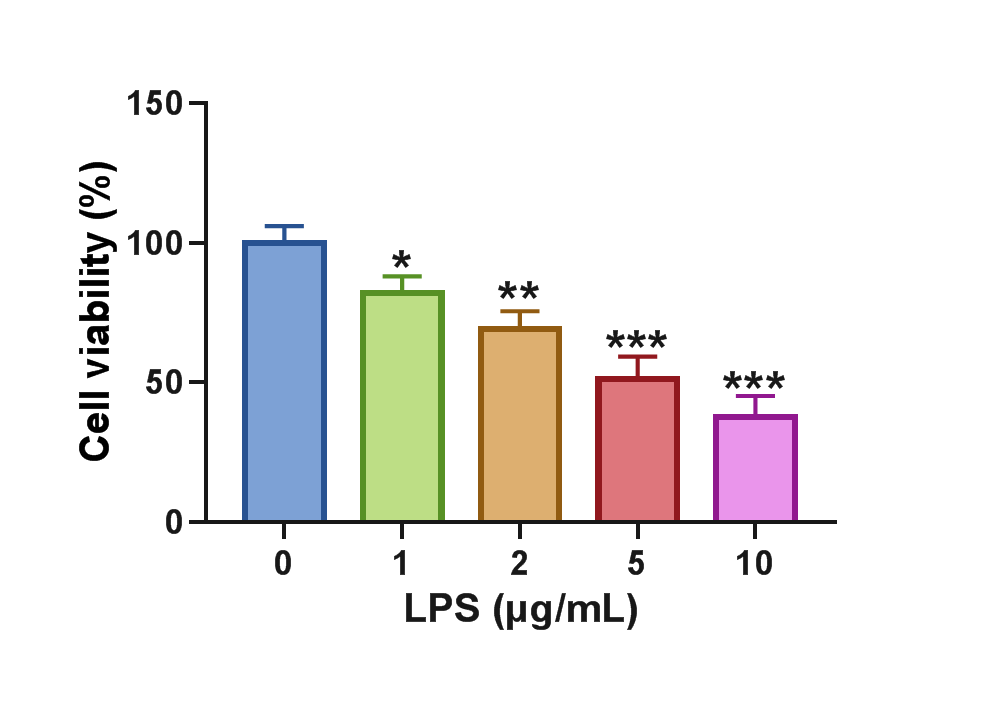

Supplement: Supplementary file 1 — Figure S1 The viability of PC12 cells after lipopolysaccharide (LPS) treatment. CCK‐8 assay for cell viability after exposing to different concentrations of LPS (0, 1, 2, 5, or 10 μg/mL) for 12 h. *p < .05, **p < .01, ***p < .001. [file IID3-11-e824-s002.tif]

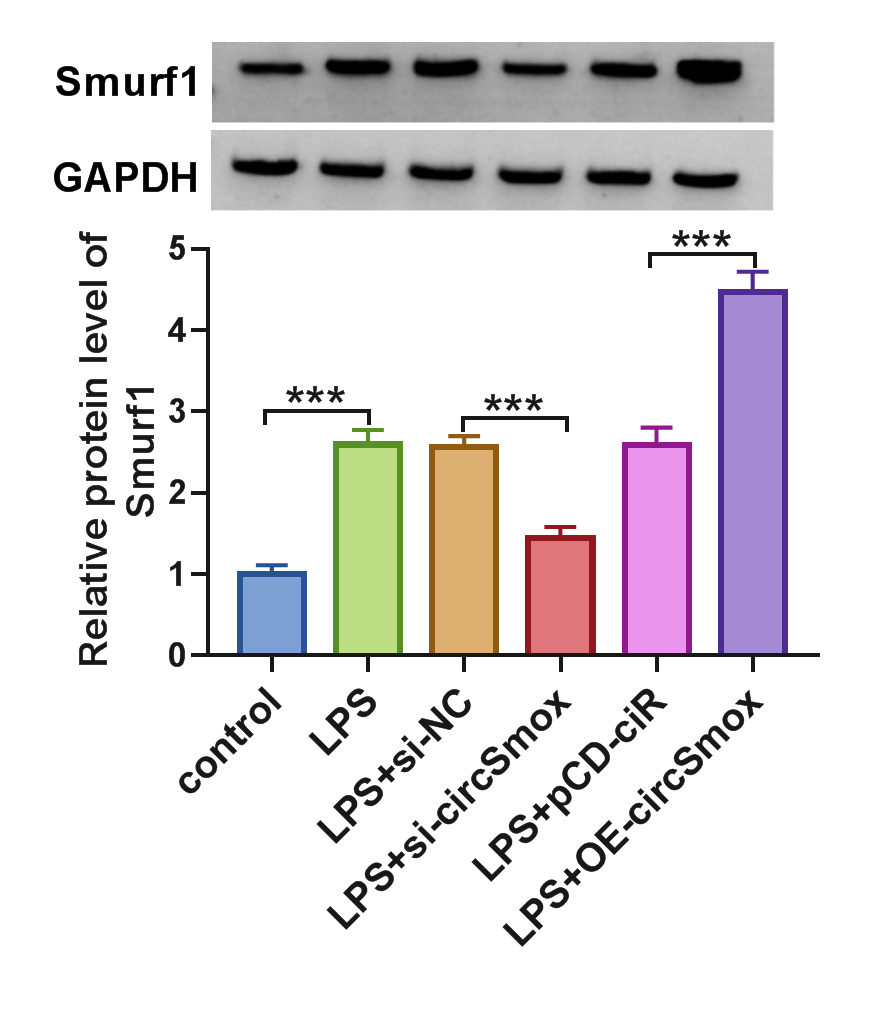

Supplement: Supplementary file 2 — Figure S2 The expression of Smurf1 in lipopolysaccharide‐treated PC12 cells after circRNA spermine oxidase overexpression or knockdown. Western blotting analysis of Smurf1 in PC12 cells after circSmox overexpression or knockdown. ***p < .001. [file IID3-11-e824-s001.tif]
